# Analysis and prediction of unplanned intensive care unit readmission using recurrent neural networks with long short-term memory

**DOI:** 10.1371/journal.pone.0218942

**Published:** 2019-07-08

**Authors:** Yu-Wei Lin, Yuqian Zhou, Faraz Faghri, Michael J. Shaw, Roy H. Campbell

**Affiliations:** 1 Department of Business Administration, University of Illinois at Urbana-Champaign, Champaign, Illinois, United States of America; 2 Department of Electrical and Computer Engineering, University of Illinois at Urbana-Champaign, Champaign, Illinois, United States of America; 3 Department of Computer Science, University of Illinois at Urbana-Champaign, Champaign, Illinois, United States of America; 4 Laboratory of Neurogenetics, National Institute on Aging, National Institutes of Health, Bethesda, Maryland, United States of America; Ben-Gurion University of the Negev, ISRAEL

## Abstract

**Background:**

Unplanned readmission of a hospitalized patient is an indicator of patients’ exposure to risk and an avoidable waste of medical resources. In addition to hospital readmission, intensive care unit (ICU) readmission brings further financial risk, along with morbidity and mortality risks. Identification of high-risk patients who are likely to be readmitted can provide significant benefits for both patients and medical providers. The emergence of machine learning solutions to detect hidden patterns in complex, multi-dimensional datasets provides unparalleled opportunities for developing an efficient discharge decision-making support system for physicians and ICU specialists.

**Methods and findings:**

We used supervised machine learning approaches for ICU readmission prediction. We used machine learning methods on comprehensive, longitudinal clinical data from the MIMIC-III to predict the ICU readmission of patients within 30 days of their discharge. We incorporate multiple types of features including chart events, demographic, and ICD-9 embeddings. We have utilized recent machine learning techniques such as Recurrent Neural Networks (RNN) with Long Short-Term Memory (LSTM), by this we have been able to incorporate the multivariate features of EHRs and capture sudden fluctuations in chart event features (e.g. glucose and heart rate). We show that our LSTM-based solution can better capture high volatility and unstable status in ICU patients, an important factor in ICU readmission. Our machine learning models identify ICU readmissions at a higher sensitivity rate of 0.742 (95% CI, 0.718–0.766) and an improved Area Under the Curve of 0.791 (95% CI, 0.782–0.800) compared with traditional methods. We perform in-depth deep learning performance analysis, as well as the analysis of each feature contribution to the predictive model.

**Conclusion:**

Our manuscript highlights the ability of machine learning models to improve our ICU decision-making accuracy and is a real-world example of precision medicine in hospitals. These data-driven solutions hold the potential for substantial clinical impact by augmenting clinical decision-making for physicians and ICU specialists. We anticipate that machine learning models will improve patient counseling, hospital administration, allocation of healthcare resources and ultimately individualized clinical care.

## Introduction

Unplanned hospital readmission is an indicator of patients’ exposure to risk and an avoidable waste of medical resources. To address the unplanned readmission issue, in 2010, the Affordable Care Act (ACA) created the Hospital Readmissions Reduction Program to penalize the hospitals whose 30-day readmission rates are higher than expected [1]. According to data released by the Centers for Medicare & Medicaid Services (CMS), since the program began on Oct. 1, 2012, hospitals have experienced nearly $2.5 billion of penalties assessed on hospitals for readmissions, including an estimated $564 million in fiscal year 2018, $144 million more than in 2016 [2].

In addition to hospital readmission, intensive care unit (ICU) readmission brings further financial risk, along with morbidity and mortality risks [3,4]. Premature ICU discharge may potentially expose patients to the risks of unsuitable treatment, which further leads to an avoidable mortality [5]. Reportedly, the mortality rates of ICU readmitted patients range approximately from 26% to 58% [6–8]. Surprisingly, even in developed countries, hospitals suffer from high ICU readmission rates, around 10% of patients will be readmitted back to ICU within a hospital stay [3]. Moreover, there is an escalating trend in the U.S. for ICU readmission rates rising from 4.6% in 1989 to 6.4% in 2003 [4]. Thus, making ICU readmission rates one of the critical quality indicators in the performance evaluation of ICU.

To reduce avoidable ICU readmission, hospitals need to identify patients with a higher risk of ICU readmission [9]. Identified patients will stay longer in the ICU and will not be exposed to readmission risks. Moreover, the additional medical resources that would have been used in unnecessary readmission can be reallocated more efficiently considering the scarcity of ICU resources compared to the general hospital. Ultimately, an efficient decision-making support system can have significant impact by assisting hospitals and ICU physicians identifying patients with high readmission probability. We can use machine learning and artificial intelligence techniques to build such decision-making support systems. Data-driven predictive models aimed at predicting ICU readmission may be built using various datasets including administrative claims [10–12], insurance claims, and electronic health records (EHRs). Among these datasets, insurance claims are not suitable for real-time prediction [13] electronic health records (EHR) have shown to provide appropriate data for medical decision-making support solutions. A systematic review of readmission prediction models [14], summarizes 26 unique readmission prediction models of which 23 models rely on EHR including the most recent work on predicting all-cause 30-day readmission by Jamei et al. [13] which proposed an accurate and real-time prediction model based on neural networks.

Even though multiple studies have developed predictive models to tackle the problem of identifying patients with a high risk of readmission, we are still far from a comprehensive practical solution. Overall, these studies have five main drawbacks. First, the scope of some predictive models is limited to a specific disease or treatment rather than a general solution. For instance solutions were focused on heart failure [15], HIV [16], diabetes [17], and kidney transplants [18]. Second, no model has been able to predict ICU readmissions to a satisfactory degree yet [19]; most models suffer from a low sensitivity of around 0.6 to 0.65 [5,13,19]. Third, most models do not utilize the sequential data structure and time series feature of many EHR parameters which can lead to information loss [20]. Last, very few attempts to understand and interpret the predictive model. Feature interpretation, as well as decision making logic, reliability, and robustness analysis of the machine learning models is crucial, and more imperative for clinical applications. This task is much more complex for deep learning techniques, which has made recent works short of explaining the decision making logic and model interpretation [21,22].

In this study, we focus on the analysis and prediction of unplanned ICU readmission using recent deep learning techniques and utilizing time series feature of data. We propose a recurrent neural network (RNN) architecture with long short-term memory (LSTM) layers to enhance the predictive model by incorporating the time series data. We also incorporate low-dimensional representations (also called embeddings) of medical concepts (e.g. diseases ICD-9 code, treatment procedure, and laboratory tests) as the input of our model [10,23]. Finally, we test, validate, and explain the proposed methods using the MIMIC-III dataset [24], containing more than 40,000 patients’ information and 60,000 ICU admission records, over a 10 year period [24]. We leverage this extensive dataset to develop predictive model which provides clinicians with the much needed decision-making support. This data-driven approach can help prevent the inappropriate discharge or transfer of patients at high-risk of ICU readmission along with reducing the associated costs and penalties.

## Methods

To accompany this report, and to allow independent replication and extension of our work, we have made the code publicly available under GPLv3 for use by non-profit academic researchers at https://github.com/Jeffreylin0925/MIMIC-III_ICU_Readmission_Analysis. The code is part of the supplemental information; it includes the step-by-step instructions of the statistical and machine learning analysis.

### Dataset construction

The readmission dataset is constructed from the MIMIC-III Critical Care Database. MIMIC-III consists of the health-related EHR data of more than 40,000 patients in the Intensive Care Units (ICU) of the Beth Israel Deaconess Medical Center between 2001 and 2012. One patient may have multiple in-hospital records in the dataset. Following the data screening process stated in [17], we first screen out the patients under age 18 and remove the patients who died in the ICU. This results in total number of 35,334 patients with 48,393 ICU stays. We then split the processed patients into training (80%), validation (10%), and testing (10%) partitions to train our model and conduct a five-fold cross-validation. Note that one patient may have multiple records, so the number of items may not equal in each fold.

To construct the dataset for ICU readmission, we categorize all selected patients and their corresponding ICU stays records into positive or negative cases. Specifically, the following cases are considered to be positive patient stays:

- 3,555 records: the patients were transferred to low-level wards from ICU, but returned to ICU again,
- 1,974 records: the patients were transferred to low-level wards from ICU, and died later,
- 3,205 records: the patients were discharged, but returned to the ICU within the next 30 days,
- 2,556 records: the patients were discharged and died within the next 30 days.

Positive cases are regarded as the ones where the patients could benefit from a prediction of readmission before being transferred or discharged. Negative cases, on the contrast, are those where the patient does not need ICU readmission. Specifically, patients who were transferred or discharged from ICU and did not return and are still alive within the next 30 days are considered to be negative cases.

### Feature extraction

In this section, we introduce the features and the time series window we use for the ICU readmission prediction task. For temporal information modeling of the time series ICU records, we use the last 48-hour data of each ICU stay. The last 48 hours before the patient is discharged or transferred are found to be the most informative data for prediction of readmission [25–26]. To cope with the problem of data missingness, we use Last-Observation-Carried-Forward (LOCF) imputation method. In cases where the last hour is missing, we include an indicator for missingness.

We use three categories of features for developing our readmission prediction model, namely chart events, ICD-9 embeddings, and demographic information of the patients. First, chart events category, which are extracted from health care provider (e.g., physicians and nurses) notes. Chart events represent the patient's' physiological conditions based on the experts' observation and opinions [19]. Second, patient variables like chronic diseases. This category has been found to strongly associate with ICU readmission risk [5,25]. Third, basic demographic information, such as gender, age, race. This category has also been demonstrated as important factors in the readmission prediction [13]. In this study, we leverage all of the above-mentioned feature categories and their time series information for the readmission prediction task. We also extract both basic and advanced statistical features from the chart events in order to compare our proposed model to traditional methods as baseline such as logistic regression.

#### Chart events

We extract 17 types of time series features from chart events within a 48-hour window. The raw features include both numerical (e.g., diastolic blood pressure) and categorical items (e.g., capillary refill rate). Details of these 17 features and their dimensions are shown in Table 1, along with their normal median value in the humans. We use the normal values later in the discussion section for machine learning model interpretation. In total 59 dimensions are constructed from the chart events; the increased number is due to the one-hot encoding of the categorical features. To identify and overcome the missing records in the chart events, we create a 17-dim binary indicator feature, appended to the chart events feature. This feature indicates whether the record for each type of chart event exists.

**Table 1:** 17 Types of features in the chart events.

Chart Events	Dim	Normal
1. Glasgow coma scale eye opening	8	4 Spontaneously
2. Glasgow coma scale verbal response	12	5 Oriented
3. Glasgow coma scale motor response	12	6 Obeys Commands
4. Glasgow coma scale total	13	15
5. Capillary refill rate	2	Normal < 3 secs
6. Diastolic blood pressure	1	70.0
7. Systolic blood pressure	1	105.0
8. Mean blood pressure	1	87.5
9. Heart Rate	1	80.0
10. Glucose	1	85.0
11. Fraction inspired oxygen	1	0.21
12. Oxygen saturation	1	97.5
13. Respiratory rate	1	15.0
14. Body Temperature	1	37.0
15. pH	1	7.4
16. Weight	1	80.7
17. Height	1	168.8

DT: Data Type, Dim: Dimension, Normal: Normal Value.

#### ICD-9 embeddings

Chronic diseases are found as one of the most important factors associated with later readmissions [25]. However, this information tends to be sparse in an EHR dataset, making them one of the most challenging to analyze with machine learning methods. In order to address the data sparsity of disease information in the EHR, we apply the approach presented in [10] to compute a pre-trained 300-dimension embedding for each ICD-9 code recorded. Utilizing a lower dimension embedding of ICD-9 benefits the model training process by avoiding a sparse representation and applying the relationship information among different diseases. For a patient with multiple diseases, we simply take the addition of embeddings of all the diseases in order to construct the feature.

#### Demographic features

The demographic features consist of the patient's' gender, age, race, and insurance type. Details of this category and its corresponding dimensions are summarized in Table 2. We include the insurance type as it could potentially influence the discharge/transfer rate. For example, although unlikely, an insurance type “uninsured” could lead to insufficient payment and might result in an unexpected discharge. In total there are 14 dimensions for the demographic category.

**Table 2:** Demographic features.

Chart Events	Dim	Option
1. Gender	2	Male/Female
2. Age	1	18–120
3. Insurance Type	5	Government, Self, Medicare, Private, Medicaid
4. Race	6	Asian, Black, Hispanic, White, Other, No Information

Dim: Dimension.

#### Statistical features for baseline models

**Fig 1:**
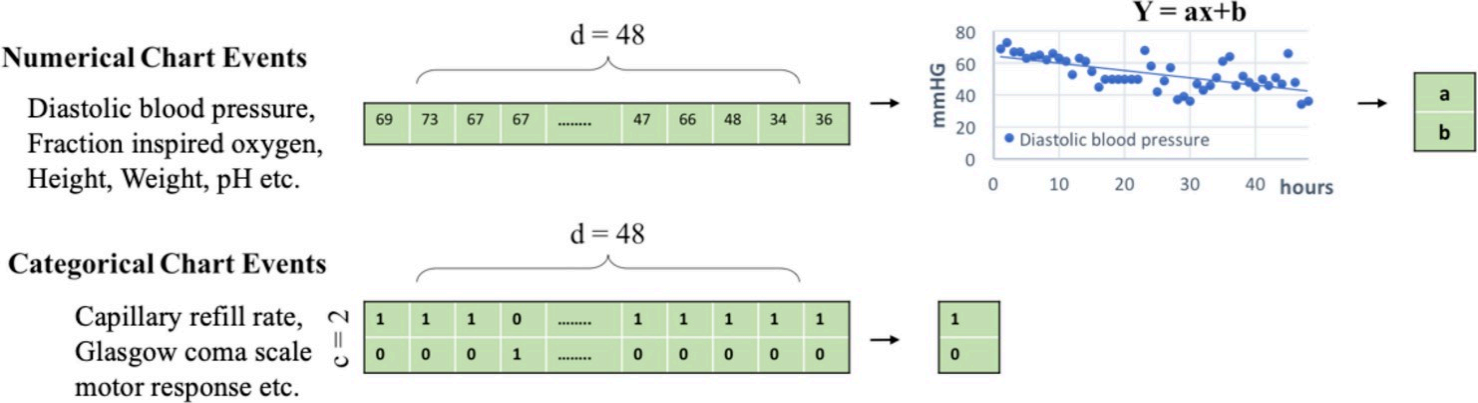
Statistical feature computation. For numerical chart events, we conduct linear regression on the 48-hour data points and record the rate and bias value as the feature. For categorical events, we simply compute the average occurrence of the categories.

For the purpose of comparison to the traditional methods, we also extract the statistical features within each 48-hour window. We include the slopes and intercepts of the regression line (*a* and *b* in *y = ax + b*) as separate features to characterize the linear trend for continuous data including the numerical chart events. Linear regression approach has been widely used in ICU readmission prediction [27–29]. For the categorical data such as capillary refill rate, we follow the approach in [30–32] to extract the mean and majority value over the total time period after transforming categorical events into binary or ordinal. Fig 1 shows an example of extracted statistical features for the baseline model comparison. After computing the statistical features, each 48-hour data window will become one single data point, resulting in 71 dimensions of chart events.

Furthermore, in order to include chart events’ volatility, we include more complex statistical features to enhance the regression model for better baseline model comparison. For numerical data, we extract: (i) quadratic term, (ii) standard deviation, (iii) mean absolute deviation, and (iv) *R*^*2*^. Adding these statistical features, results in the increase of dimensions from 2 to 6 for numerical features. For categorical data, we extract: (i) majority value, and (ii) how often the value switches. These statistical features enable us to better capture the volatile nature of ICU events in the traditional baseline models. We call the earlier statistics “basic statistical features (B-STAT)” and the combination of basic and more complex statistical features “advanced statistical features (A_STAT)” for the rest of this paper.

### Machine learning model structure

#### Baseline models

The first baseline model that we include is the logistic regression models. In this study, we implement logistic regression with both L1 and L2 regularization penalty. We further train three conventional machine learning models as our baseline, including Naive Bayes, Random Forest, and Support Vector Machines (SVM).

#### Convolutional neural network (CNN) model

**Fig 2:**
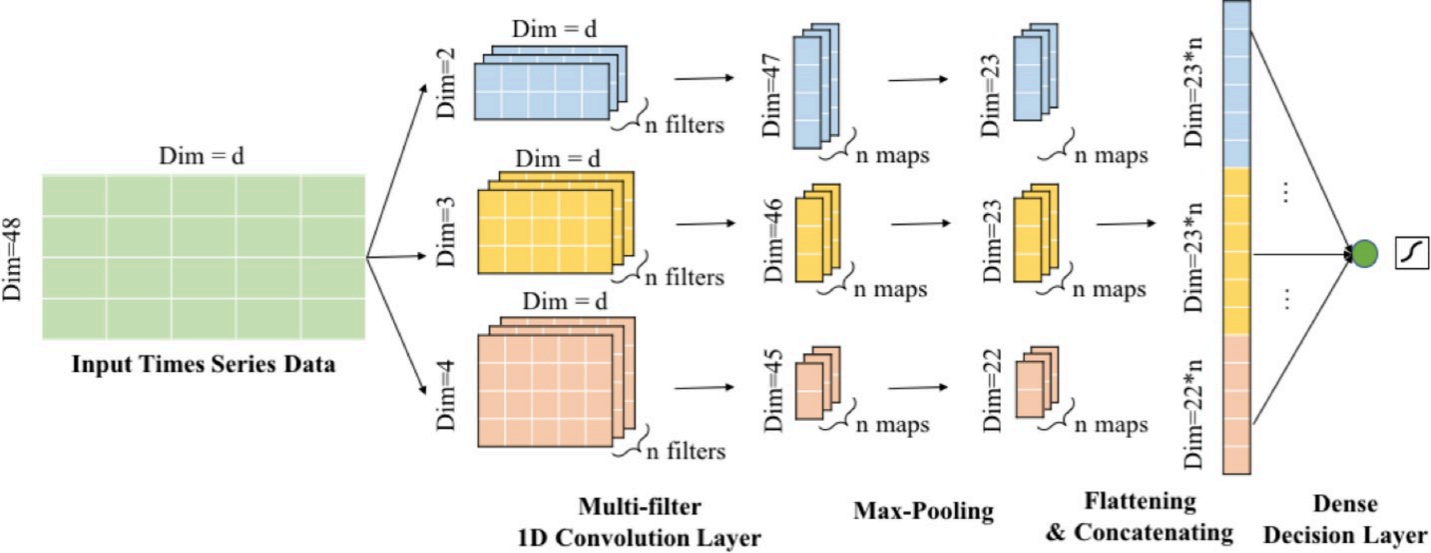
The 1D multi-filter convolutional neural network. We conduct the convolution on the time axis with 48-hour time window and D dimension using filter size 2, 3 or 4 accordingly. The computed feature maps are finally concatenated and fully connected to a dense decision layer with one output neuron.

**Fig 3:**
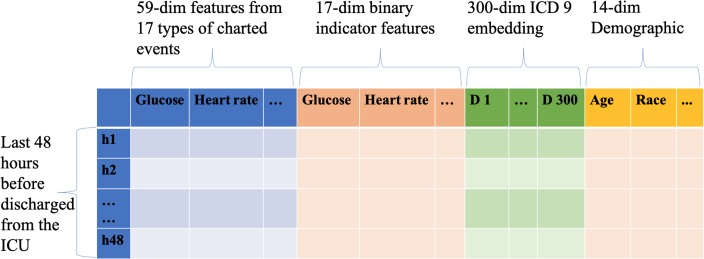
The data structure of input data used with CNN and LSTM models. D: dimension, h: hour.

We also implement a CNN-based model for comparison to our LSTM model. CNN-based models are found useful in analyzing longitudinal EHR data [21]. Shown in Fig 2, we use a multi-filter CNN structure introduced in [33]. We use the CNN model on a comprehensive and longitudinal representation of data with 18,720 dimensions as shown in Fig 3. We conduct the convolution on the time axis with 48-hour time window and *D* dimension using filter size 2, 3 or 4 accordingly. The computed feature maps are finally concatenated and fully connected to a dense decision layer with one output neuron.

#### Long short-term memory (LSTM) model

**Fig 4:**
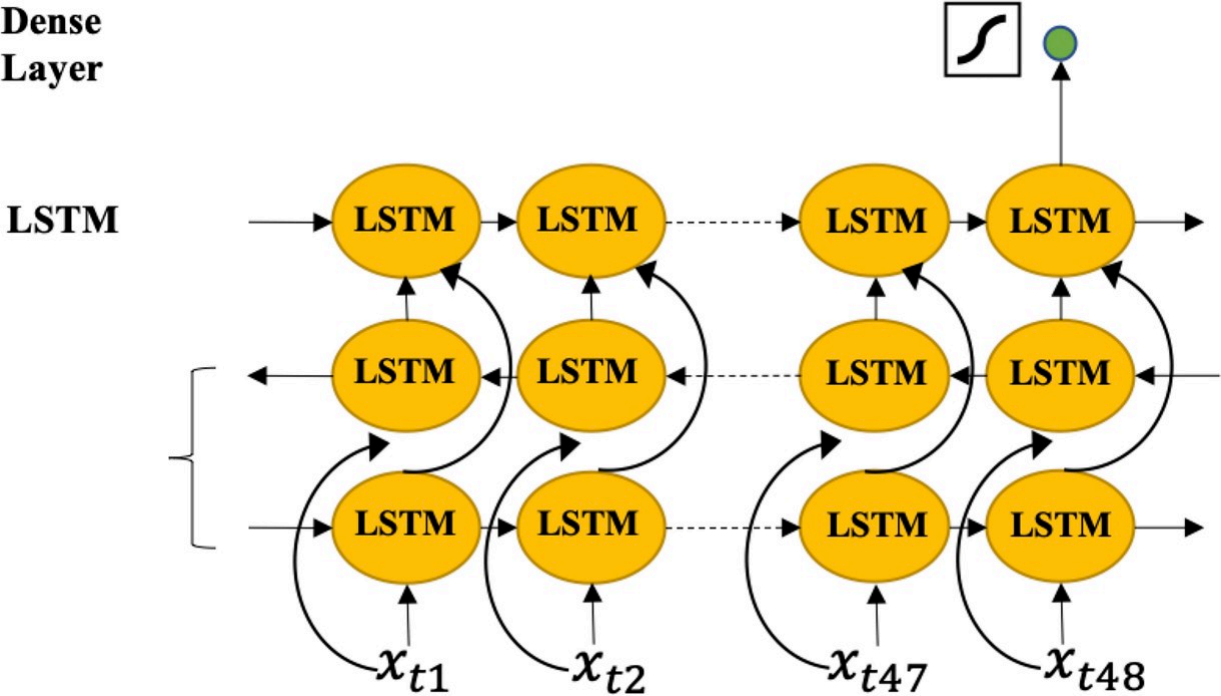
LSTM model. A bidirectional LSTM combined with an additional LSTM layer, followed by a dense decision layer with one output neuron activated by a sigmoid function. Overall, we have 16 hidden units in our LSTM layer.

LSTM networks are found well-suited to making predictions based on time series data, especially for clinical measurements where there can be lags of unknown duration and missing values in a time series [34]. Fig 4 shows our utilized LSTM model. We use a bidirectional LSTM combined with an additional LSTM layer, followed by a dense decision layer with one output neuron activated by a sigmoid function. Overall, we have 16 hidden units in our LSTM layer. Bidirectional LSTM learns the temporal information across the whole training window. Considering an ICU stay record with a length of 48 hours, observation at each hour is denoted by *x*_*t*_ *∈ R*^*1×D*^, where *t* is an integer from 1 to 48, and *D* is the feature dimension size. The output of a single LSTM cell can be computed by the following equations,

$$i_{t}=\sigma (W_{}\cdot [h_{t‐1},x_{t}]+b_{i})$$

$$f_{t}=\sigma (W_{f}\cdot [h_{t‐1},x_{t}]+b_{f})$$

$${\hat{C}}_{t}=\tanh\;\left(W_{C}\cdot [h_{t‐1},x_{t}]+b_{c}\right)$$ (1)

$$C_{t}=f_{t}⊙C_{t‐1}+i_{t}⊙{\hat{C}}_{t}$$

$$o_{t}=\sigma (W_{o}\cdot [h_{t‐1},x_{t}]+b_{o})$$

$$h_{t}=o_{t}⊙\tanh\left(C_{t}\right)$$

The above functions can be simply denoted by *h*_*t*_ *= LSTM(h*_*t-1*_ *; x*_*t*_*)*. We utilize the hidden value of the last time stamp to predict the readmission possibility, thus the final output after going through the dense layer would be,

$$r_{T}=\sigma (W_{r}\cdot h_{48}+b_{r})$$ (2)

where σ is the indicator of the sigmoid activation function, and the *r*_*T*_ represents the prediction probability of whether this patient with the ICU stay record will be readmitted, ranging from zero to one. The dimension of *h*_*t*_ is *R*^*1×16*^, therefore the *W*_*r*_ *∈ R*^*16×1*^. We also use binary cross entropy loss to update the weights. In addition to separate CNN and LSTM based models, we also implement and compare the performance of the LSTM and CNN combination models. We implemented all the models using Keras based on the benchmark code of [20]. The learning rate of training was set to 1e^−3^, and we used Adam optimizer to train the model with beta 0.9. We trained at most 50 epochs and selected the model with the highest AUC on the validation partition following the logic in [34]. During the evaluation, we set up the decision threshold as 0.5.

We evaluate the performance of the predictive models by performing a five-fold cross-validation and measuring the area under the receiver operating curve (AUC) generated by plotting sensitivity vs 1 − specificity. We use cross-validation for detecting and preventing possible overfitting or selection bias. We randomly divided the dataset into five subsamples, retained a single subsample as the validation data for testing the model, and the remaining four samples used as training data. We repeated the process five times (the folds), with each of the subsamples used exactly once as the validation data. Performance of the model in each fold was measured and then results from all five folds were averaged to produce a single estimation for the model’s performance.

AUC measures the overall performance of the recall with respect to different false positive rate. Models with higher AUC will demonstrate a more powerful screening capability in assisting the physicians. In order to further evaluate the machine learning models for a clinical setting, we assess the AUC along with operating points corresponding to high-sensitivity (true positive rate) and high-specificity (true negative rate) of the algorithm with respect to the reference standard [35–39]. Targeted operating points are used for different clinical purposes, for instance high-sensitivity is targeted for ruling out the disease, whereas high-specificity is used for ruling in the disease [40]. In this study, in order to evaluate the performance under consistent conditions, the operating points correspond to fixed sensitivity and specificity at 0.80 and 0.85 [35,37,38]. In practice, high-sensitivity (or recall rate of positive cases) plays a more important role in screening the patients. In essence, a highly sensitive test indicates that the model can correctly identify patients with a high risk of readmission in a critical department such as ICU.

## Results

In this section, we illustrate the experiments we conducted to evaluate the performance of the predictive models. We evaluate the conventional models (logistic regression, random forest, Naive Bayes, and SVM), as well as, the deep learning based CNN and temporal LSTM models. We compare and obtain the optimal ICU readmission prediction solution.

### Baseline models

We first evaluate logistic regression models with both *L1* and *L2 regularization* penalty. Results are shown in Table 3 part (a) under “Baseline–Regression”. We first observe that using logistic regression with L2 regularization on the advanced statistical features (A_STAT) can slightly improve the AUC performance compared to the basic statistical features (B_STAT), 0.770 (95% CI, 0.758–0.782) to an AUC of 0.771 (95% CI, 0.759–0.783). However, we do not observe any AUC improvement for the logistic regression with L1 regularization by having more advanced statistical features (stays at AUC of 0.775 (95% CI, 0.765–0.786)). In addition to advanced statistical features, the demographic features can also slightly improve the performance from an AUC of 0.771 (95% CI, 0.759–0.783) to an AUC of 0.773 (95% CI, 0.762–0.787) using the logistic regression with L2 regularization.

**Table 3:** Performance comparison of various machine learning models on different sets of features.

Model	Features	Re-1 (95% CI)	A.R (95% CI)
**(a) Baseline—Regression**			
LR-L2	L48-h B_STAT + ICD9	0.670 (0.647–0.694)	0.770 (0.758–0.782)
LR-L2	L48-h A_STAT + ICD9	0.670 (0.648–0.692)	0.771 (0.759–0.783)
LR-L2	L48-h A_STAT + ICD9 + D	0.676 (0.650–0.703)	0.773 (0.762–0.787)
LR-L1	L48-h B_STAT + ICD9	0.669 (0.647–0.691)	0.775 (0.764–0.786)
LR-L1	L48-h A_STAT + ICD9	0.669 (0.656–0.681)	0.775 (0.765–0.786)
LR-L1	L48-h A_STAT + ICD9 + D	**0.680** **(0.662–0.697)**	**0.777** **(0.765–0.789)**
**(b) Baseline–Conventional Machine Learning**			
NB	L48-h B_STAT + ICD9 + D	0.453 (0.434–0.472)	0.709 (0.702–0.716)
NB	L48-h A_STAT + ICD9 + D	0.509 (0.479–0.540)	0.706 (0.698–0.713)
RF	L48-h B_STAT + ICD9 + D	0.563 (0.548–0.578)	0.714 (0.703–0.725)
RF	L48-h A_STAT + ICD9 + D	0.565 (0.550–0.580)	0.712 (0.693–0.730)
SVM	L48-h B_STAT + ICD9 + D	0.701 (0.686–0.715)	0.775 (0.765–0.785)
SVM	L48-h A_STAT + ICD9 + D	**0.703** **(0.685–0.720)**	**0.779** **(0.768–0.789)**
**(c) Feature Selection**			
LSTM	F48-h CE + ICD9	0.731 (0.723–0.740)	0.777 (0.769–0.785)
LSTM	L48-h CE + ICD9	0.717 (0.692–0.742)	0.784 (0.772–0.795)
LSTM	L48-h CE	0.593 (0.537–0.649)	0.704 (0.697–0.710)
LSTM	L48-h CE + ICD9 + D	**0.733** **(0.698–0.768)**	**0.787** **(0.771–0.802)**
**(d) Model Selection**			
CNN	L48-h CE + ICD9	0.665 (0.586–0.745)	0.780 (0.774–0.786)
CNN	L48-h CE + ICD9 + D	0.735 (0.676–0.794)	0.784 (0.773–0.794)
CNN+LSTM	L48-h CE + ICD9	0.739 (0.670–0.807)	0.785 (0.775–0.795)
CNN+LSTM	L48-h CE + ICD9 + D	0.710 (0.648–0.771)	0.787 (0.775–0.799)
LSTM+CNN	L48-h CE + ICD9	0.729 (0.647–0.811)	0.786 (0.776–0.796)
LSTM+CNN	L48-h CE + ICD9 + D	**0.742** **(0.718–0.766)**	**0.791** **(0.782–0.800)**

Acc: Accuracy. Pre: Precision. Re: Recall. A.R: AUC under ROC. A.P: AUC under PRC. L48: Last 48 hours. F48: First 48 hours. CE: Chart Events. D: Demographic features. C.I.: 95% confidence interval. B_STAT (basic statistical features): slope and intercept. A_STAT (advanced statistical features): B_STAT plus continues and categorical features including quadratic term, standard deviation, mean absolute deviation, R^2^, Majority value, value change frequency.

Overall, we see that the prediction accuracy can be slightly improved by adding more complex statistical features as well as demographic ones. The best performing logistic regression model is with L1 regularization on A_STAT combined with the demographic features, AUC of 0.777 (95% CI, 0.765–0.789) and sensitivity of 0.680 (95% CI, 0.662–0.697). Furthermore, we trained three conventional machine learning models as our baseline, including Naive Bayes, Random Forest, and SVM on both B_STAT and A_STAT features. The results are shown in Table 3, part (b), “Baseline–Conventional Machine Learning”. SVM outperforms other traditional methods by reaching an AUC of 0.779 (95% CI, 0.768–0.789) with A_STAT, which is a negligible increase from an AUC of 0.775 (95% CI, 0.765–0.785) with B_STAT.

### CNN and LSTM models

We first conduct a feature ablation study to evaluate the effect of various feature selections on the system’s performance. Then, we attempt multiple model structures including bidirectional LSTM, CNN, and the combinations of both.

#### Feature selection

We select the Bidirectional LSTM as our base model and deploy different combinations of feature inputs. As shown in Table 3 part (c), our results demonstrate that the last-48h features perform relatively better than the first-48h data in terms of positive recall rate and AUC. In addition, ICD-9 embedding is necessary for predicting the readmission rate. We also observe that the demographic features greatly benefit the performance. Overall, the full set of features including Last-48h chart events and their identifiers, ICD-9 embeddings, and demographic information perform the best among all the combinations.

#### Model selection

**Fig 5:**
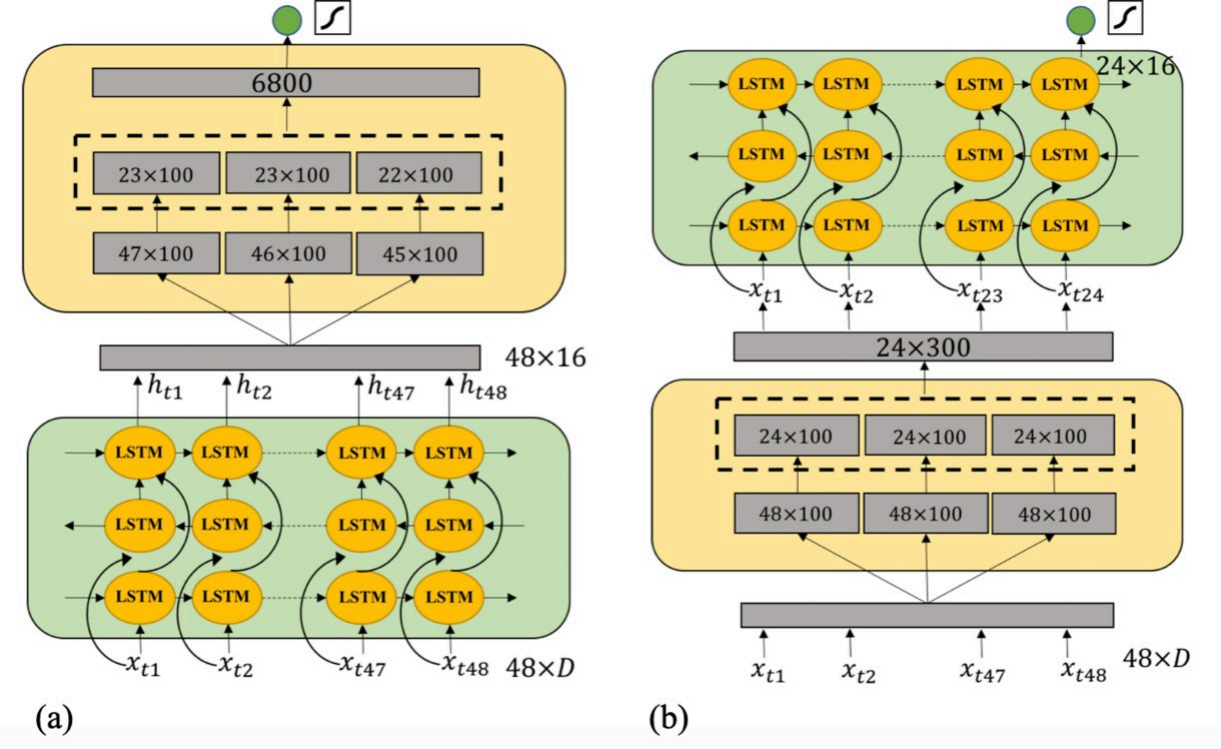
Combination of LSTM and CNN models. (a) CNN+LSTM model, the CNN follows a multi-filter convolution computation with zero padding to maintain the timestamp consistency for different groups of feature maps. The following LSTM only outputs the hidden units of the last time stamp. (b) LSTM+CNN model, CNN computes the feature maps without zero padding after receiving the output hidden unit sequence from LSTM.

We attempted multiple model structures including bidirectional LSTM, CNN, and the combinations of both. Fig 5 shows our strategy for combining the bidirectional LSTM and CNN models.

**Fig 6:**
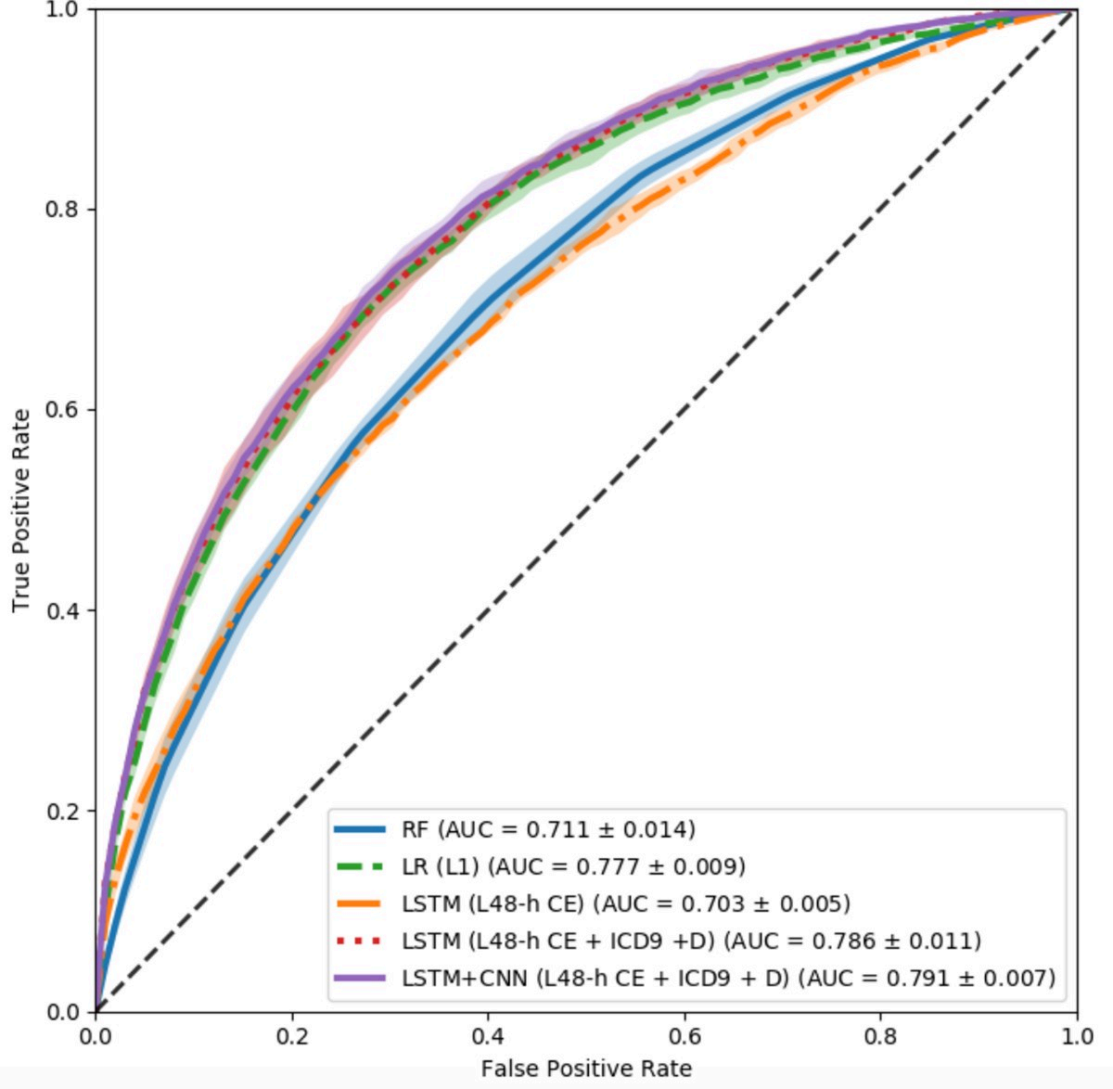
ROC curve of selected high performing machine learning models. The color bar is the error bar of the ROC curve with five-fold cross-validation. LSTM-CNN model performs relatively better than other ones. CE: chart events. D: Demographic features.

We use the 1D multi-filter CNN model introduced in the previous section. As for the CNN+LSTM model, the CNN follows a multi-filter convolution computation with zero padding to maintain the timestamp consistency for different groups of feature maps. The following LSTM only outputs the hidden units of the last time stamp. However, for the LSTM+CNN model, CNN computes the feature maps without zero padding after receiving the output hidden unit sequence from LSTM. As shown in Table 3 part (d), our experimental results reveal that LSTM followed by a CNN, utilizing all the feature sets, obtains a higher positive recall rate and overall prediction performance. The proposed model outperforms the conventional machine learning approaches trained on both basic and advanced statistical features. The ROC curve for some of the selected high performing machine learning models are shown in Fig 6.

To further demonstrate the ability of deep learning model in the readmission prediction, we look at the operating points corresponding to high-sensitivity (true positive rate) and high-specificity (true negative rate) of the algorithm. Table 4 summarizes the performance of the algorithms. Using the operating cut point with high specificity of 0.85 and 0.8, we observe that LSTM+CNN results in the highest sensitivities of 0.548 (95% CI, 0.522–0.575) and 0.619 (95% CI, 0.597–0.642) respectively, a significant improvement from the best baseline. Evidently, even the basic LSTM model outperforms the best baseline, regression with L1 regularization, by improving the sensitivities from 0.525 (95% CI, 0.505–0.546) to 0.540 (95% CI, 0.503–0.577) and 0.596 (95% CI, 0.575–0.618) to 0.611 (95% CI, 0.573–0.649) respectively.

**Table 4:** Performance comparison of machine learning models at high-sensitivity and high-specificity operating points.

Model	Re-1 (95% CI) (Re-0 fixed near 0.85)	Re-1 (95% CI) (Re-0 fixed near 0.8)	Re-0 (95% CI) (Re-1 fixed near 0.85)	Re-0 (95% CI) (Re-1 fixed near 0.8)
**Baseline—Regression**				
LR-L2	0.507 (0.491–0.522)	0.590 (0.570–0.611)	0.516 (0.484–0.549)	0.596 (0.563–0.629)
LR-L1	**0.525** **(0.505–0.546)**	**0.596** **(0.575–0.618)**	**0.518** **(0.476–0.561)**	**0.599** **(0.573–0.626)**
**Baseline–Conventional Machine Learning**				
NB	0.358 (0.333–0.383)	0.468 (0.447–0.489)	0.247 (0.238–0.256)	0.330 (0.318–0.342)
RF	0.402 (0.364–0.439)	0.475 (0.445–0.504)	0.415 (0.381–0.449)	0.487 (0.457–0.516)
SVM	**0.519** **(0.498–0.539)**	**0.596** **(0.584–0.608)**	**0.532** **(0.498–0.565)**	**0.608** **(0.588–0.628)**
**Deep Learning**				
LSTM	0.540 (0.503–0.577)	0.611 (0.573–0.649)	0.532 (0.503–0.561)	0.608 (0.590–0.626)
CNN	0.531 (0.513–0.549)	0.604 (0.579–0.630)	0.527 (0.493–0.561)	0.607 (0.573–0.641)
CNN+LSTM	0.543 (0.510–0.576)	0.617 (0.590–0.644)	0.535 (0.515–0.556)	0.611 (0.591–0.632)
LSTM+CNN	**0.548** **(0.522–0.575)**	**0.619** **(0.597–0.642)**	**0.537** **(0.515–0.559)**	**0.618** **(0.593–0.643)**

We then evaluated a second operating point for the algorithm, with a high-sensitivity, reflecting an output that would be used for a screening tool. Using this operating point, LSTM+CNN had sensitivities of 0.85 and 0.8 and the highest specificities of 0.537 (95% CI, 0.515–0.559) and 0.618 (95% CI, 0.593–0.643), again an improvement from conventional machine learning models.

## Discussion

In this section, we dive deeper into our machine learning model in an effort to further interpret the results, its capabilities, and limitations. We perform ablation study to investigate the most important factors that the deep learning model has learned in order to predict the ICU readmission. Then, we review the clinical literature for additional verification and a better clinical understanding of the deep learning model. Finally, we examine the advantages and strength of the proposed model over traditional machine learning models. We look at the characteristics and statistics for the true positive sets of each model.

### Model interpretation: Feature ablation test

**Fig 7:**
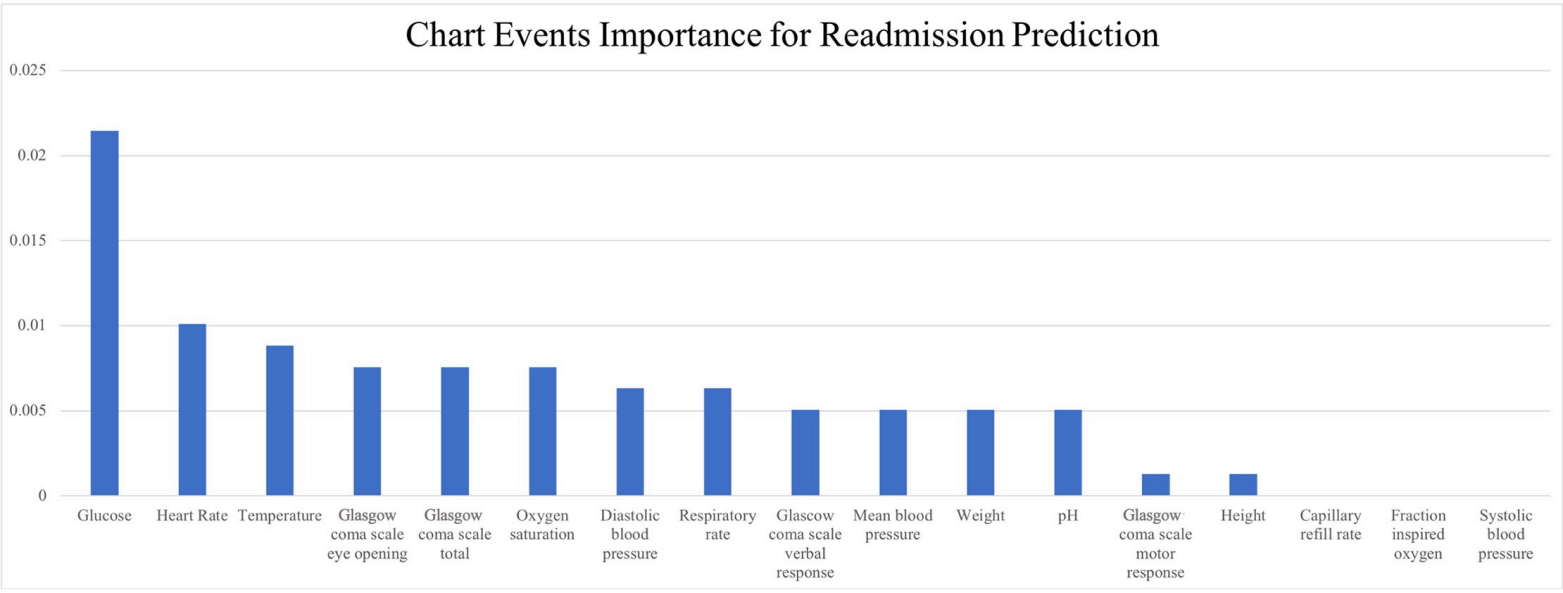
The results of feature ablation test. **The importance of chart events for predicting the ICU readmission.** The y-axis shows the changing ratio of the prediction results after we replace the original feature with its normal value.

We conducted the feature ablation test on the chart events to better understand the underlying logic of our proposed model. We selected all the positive cases from the testing partition. Then we obtained all the true positive samples through running the LSTM+CNN model utilizing all the features. These true positive cases are the ones recalled correctly by our proposed model. For each case, we iterated over all the chart events, each time, changing only one event to its normal value in the humans. We recorded the number of cases that were falsely predicted due to the change. Then we ranked all the chart events according to the change numbers. Fig 7 shows the results of feature ablation test based on the changing ratio of the prediction results after we replace the original feature with its normal value. We see that Glucose is the most important factor learned by the deep learning model for the readmission prediction task, while Capillary Refill Rate, Fraction inspired Oxygen, and Systolic Blood Pressure do not have significant influence on the prediction results. However, the performance change of the predictive model is not dramatic. We believe this may be due to possible biological and clinical correlation among different factors. This can be further evaluated by the back-propagation approach in future work.

### Model interpretation: Features in line with the clinical literature

Furthermore, we review the clinical literature for additional verification and a better understanding of the deep learning model system. The results of the feature ablation test from the previous section point out that abnormal Glucose, Heart Rate, Body Temperature, Glasgow Coma Scale, and Oxygen Saturation are the top five important features in predicting unplanned readmission in the ICU. Interestingly, the underlying deep learning logic and its findings are in line with the existing clinical literature. Prior research has found that the presence of comorbidities, such as diabetes, heart failure, renal failure, and pneumonia, are the main risk factors resulting in unplanned readmissions [41,42]. These disorders are shown to have strong correlations with abnormal features identified by our model [17]. Moreover, several studies have worked on the readmission problem by only focusing on the aforementioned conditions.

For instance, many researchers have focused on hospitalization and unplanned readmissions by looking at the abnormal Glucose status. Berry et al discovered the significant positive relationship between levels of admission blood glucose and risk of readmission for patients with heart failure [43]. Evans et al identified that the glucose level on admission performs as a prognostic predictive factor for early readmission rates, even for those with diabetes [44]. Dungan has demonstrated that higher time-weighted mean glucose is associated with the increase of congestive heart failure (CHF) readmission [45]. Emons et al focused on hypoglycemia-related readmission issue and expose the linear relationship between blood glucose level closest to discharge and the risk of hypoglycemic readmission [46].

Heart failure is another main risk factor resulting in early readmission [47]. Heart failure indicates that the cardiac muscle cannot pump the blood properly. This behavior is strongly reflected through abnormal heart rate [48,49]. Keenan et al developed a hierarchical logistic regression model to predict readmission for those patients hospitalized with heart failure issues [48]. Hammill et al utilize heart rate record during hospitalization as one of the main features to predict 30-day outcomes after heart failure hospitalization [49].

In addition, patients with renal failure are suggested to be among the highest risk patients with 30-day readmission [50]. Previous studies have shown that body temperature is a vital determinant of ischemic renal injury [51]. Moreover, Sood et al found that body temperature and Glasgow coma scale are two significant features to predict early ICU readmission for patients with end-stage renal disease (ESRD) [52].

Last but not least, a study has revealed that around 140,000 hospital readmissions per year are owing to pneumonia [53]. Halm et al apply a regression to examine the relationship between patients’ instabilities and the risk of early readmission. They proposed a list of unstable factors leading to higher risk of 30-day hospital readmission, including (temperature >37.8°C, heart rate >100 bpm, respiratory rate >24/min, systolic blood pressure <90 mmHg, oxygen saturation <90%, inability to maintain oral intake, and abnormal mental status) [54].

In summary, the underlying logic of our deep learning model, as well as the most important features identified by the model, are in line with the existing clinical literature.

### Strengths of the model

To better understand the advantages and strength of the LSTM-based model over the traditional models, we investigate the positive patients correctly predicted by the LSTM+CNN but misclassified by the logistic regression with L1 regularization. Overall, there are 441 positive patients, across all the testing partition folds, who are correctly predicted only by the LSTM+CNN model and not the logistic regression. We refer to these 441 patients as LSTM-C set. Meanwhile, 3,068 cases are correctly predicted by both the LSTM+CNN and Logistic Regression with L1 regularization. We refer to these 3,068 cases as LSTM-LR-C set.

LSTM-based models are found to provide a robust prediction for time series with notable fluctuations in the data [55]. We verify this phenomenon by measuring the degree of value oscillation for LSTM-C and LSTM-LR-C, and also looking at individual cases. We introduce *D*_*nm*_, measuring the degree of oscillation for record *n* of chart event *m*. Given a numerical chart event sequence *E*_*nm*_ = {*x*_*t*_}, where *t ∈* [1; 48], then *D*_*nm*_ can be computed by,

$$D_{nm}=\frac{1}{T-1}\sum_{t=2}^{T}\left|x_{t}-x_{t-1}\right|$$ (3)

where *T* is equal to the length of a record, normally 48, if there is no missing data.

Using *D*_*nm*_ as a measure for the degree of oscillation, we compute the highest oscillation for each stay across all the 12 numerical chart events and compare their statistical distributions in LSTM-C and LSTM-LR-C. We first estimate *P*_*m*_, the cumulative density function (CDF) of each chart event on the whole positive set. Then we remapped each *D*_*nm*_ to the probability *p*_*nm*_, and computed the maximum probability *w*_*n*_ for each record *n* by,

$$p_{\mathrm{nm}}=P_{m}(D_{\mathrm{nm}})$$ (4)

$$w_{n}=\underset{m}{\max}\;p_{\mathrm{nm}}$$ (5)

where *w*_*n*_ represents the highest oscillation among all the chart events for this record.

**Fig 8:**
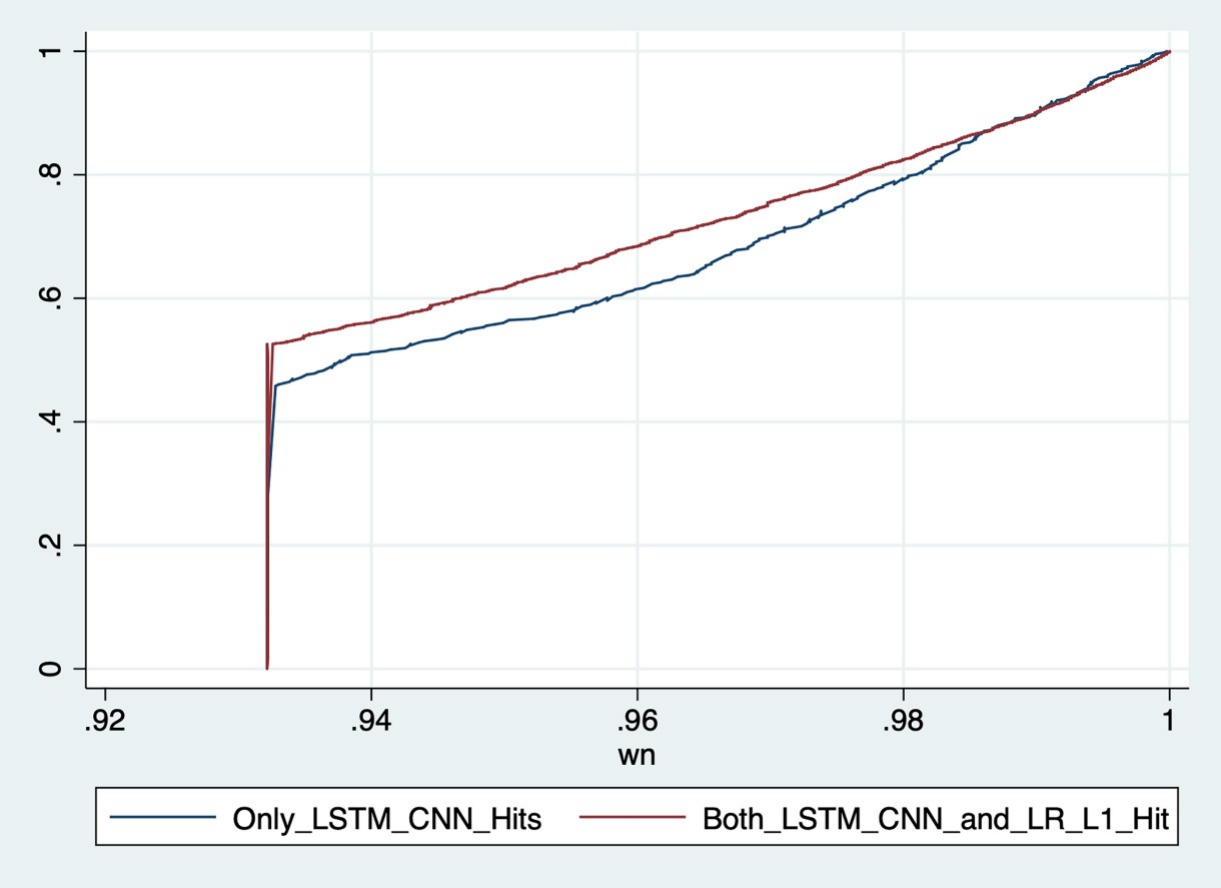
**Cumulative density function curve of LSTM-LR-C (red line) and LSTM-C (blue line)**. Figure shows that there are more patient records in the LSTM-C which have at least one chart event with high oscillation sequence. Essentially, compared to Logistic Regression, our LSTM+CNN model is capable of capturing high volatile time series behavior, a common pattern in high-risk ICU patients.

Finally, for both LSTM-C and LSTM-LR-C sets, we plotted the CDFs of the estimated histograms of *w*_*n*_ in Fig 8. We can see that there are more patient records in the LSTM-C which have at least one chart event with high oscillation sequence. Essentially, compared to Logistic Regression, our LSTM+CNN model is capable of capturing high volatile time series behavior, a common pattern in high-risk ICU patients.

**Fig 9:**
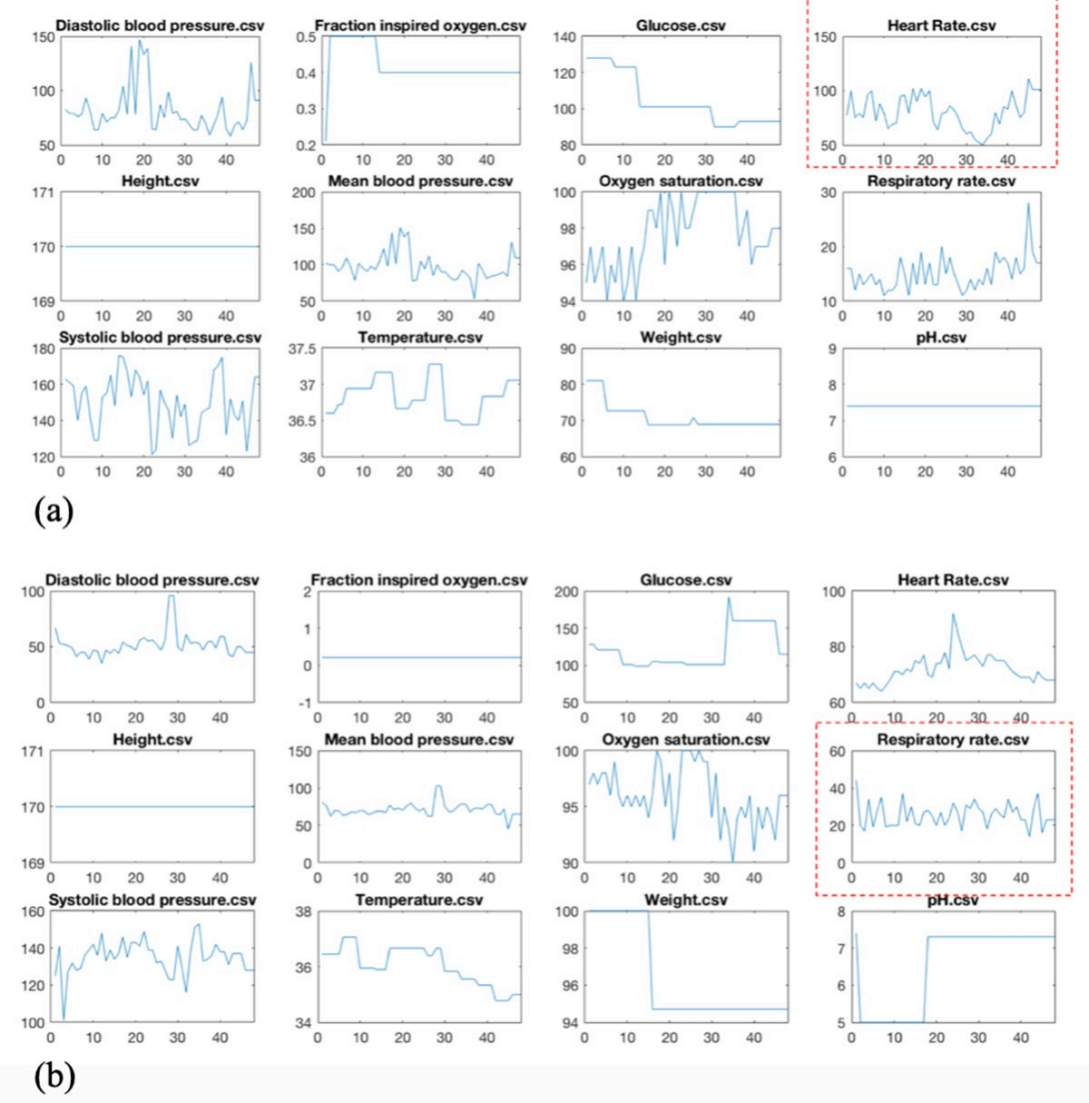
**(a) A selected ICU stay with the highest heart rate event oscillation, and (b) another case with the highest oscillation of respiration rate.** These two patients are predicted correctly by the LSTM-CNN model, but wrongly by the traditional models. In both cases, the abnormal sequence has oscillated around the normal value of the chart event, which in return a linear model would regress it to a normal value with a negligible slope. Effectively, our LSTM-CNN is capable of capturing such high volatile behavior, a common pattern among high-risk ICU patients with unstable status.

To further study the strength of our LSTM+CNN solution, we look at individual cases. For each chart event, we selected the patients with the highest *D*_*nm*_ in the LSTM-C and plotted the sequence values of their stay. Fig 9 illustrates two of these patients. Both patients have high volatile chart events. However, in both cases the abnormal sequence has oscillated around the normal value of the chart event, which in return a linear model would regress it to a normal value with a negligible slope. Effectively, a linear model would lose a very important factor in predicting the readmission: repeated illness and unstable status.

We further investigate the strength and weaknesses of the LSTM-based model by looking at the oscillation issue among all the chart events. We investigate the differences between positive patients who are predicted correctly only by the logistic regression with L1 regularization and those who are predicted correctly only by the LSTM+CNN. As mentioned earlier, there are 441 positive patients who are predicted correctly only by the LSTM+CNN model, denoted as the LSTM-C set. On the other hand, there are 147 cases that are predicted correctly only by the logistic regression with L1 regularization, we denote this set by LR-C.

Our goal is to identify the differentiating factors between the LR-C set and the LSTM-C. We analyze the fluctuation distribution for each chart event in both sets. We use the Eq (3) to calculate the *D*_*nm*_, measuring the degree of oscillation for chart event *m* of patient *n*. We then estimated the cumulative density function (CDF) of each chart event in each set. For each chart event, we conduct Kolmogorov–Smirnov test (K-S test) on factor *D*_*nm*_ to compare the distributions of this factor between the two sets. The results are shown in Table 5.

**Table 5:** Kolmogorov–Smirnov (K-S) test for the distribution of fluctuation between LSTM-C and LR-C for each chart event.

Two-sample Kolmogorov–Smirnov (K-S) test		
	D	P value
Glucose	0.1519	0.012
Heart rate	0.0635	0.766
Temperature	0.0839	0.420
Oxygen saturation	0.1678	0.004
Diastolic blood pressure	0.0794	0.491
Respiratory rate	0.1020	0.201
Mean blood pressure	0.0680	0.687
Weight	0.0476	0.964
pH	0.0635	0.766
Height	0.0181	1.000
Fraction inspired oxygen	0.0499	0.947
Systolic blood pressure	0.0635	0.766

**Fig 10:**
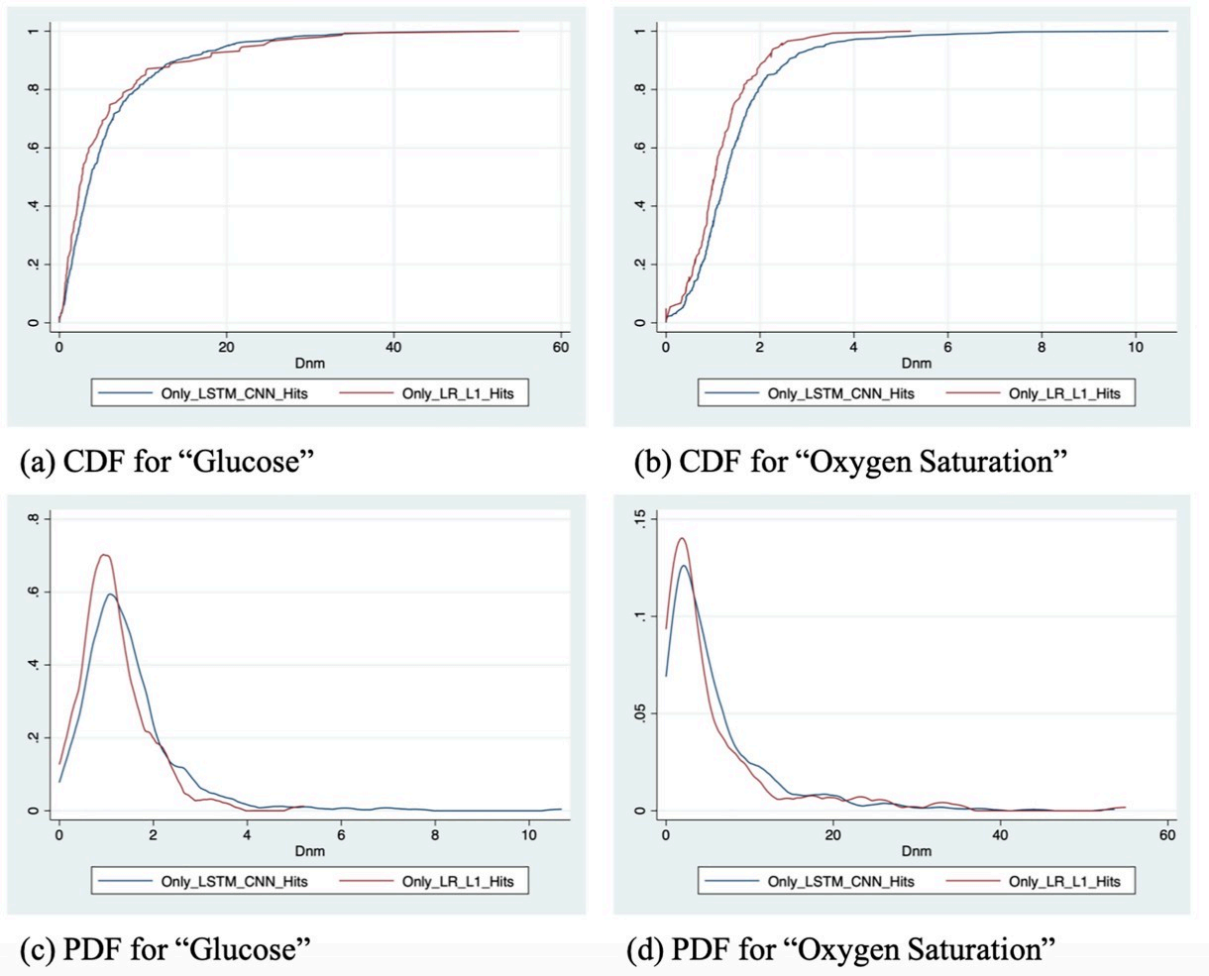
Cumulative density function (CDF) plots and probability density function (PDF) plots.

Results reveal that patients in the LR-C tend to have a higher probability of achieving lower scores of factors *D*_*nm*_ on “Glucose” than patients in the LSTM-C (maximal absolute difference between the distribution functions (D) = 0.1519, p-value = 0.012). In addition, we also observe that patients in the LR-C set tend to have a higher probability of achieving lower scores of factors *D*_*nm*_ on “Oxygen Saturation” than patients in LSTM-C (D = 0.1678, p-value = 0.004). The CDF of “Glucose” and “Oxygen Saturation” are shown in Fig 10 part (a) and (b). We further use the Probability density function (PDF) plots of both features to show this phenomenon in Fig 10 part(c) and (d). The results in this section further enhance the suggestion that deep learning has advantages over logistic regression in predicting datasets with large fluctuation of time series features, “Glucose” and “Oxygen Saturation” in this case.

### Comparison with the baseline models

In addition to comparing LSTM-based model and logistic regression with L1 regularization, we further compare LSTM-based model with all the six baseline regression models, including: (i) L1 logistic regression with B-STAT, (ii) L1 logistic regression with A_STAT, (iii) L1 logistic regression with A_STAT and Demographic features, (iv) L2 logistic regression with B-STAT, (v) L2 logistic regression with A_STAT, and (vi) L1 logistic regression with A_STAT and Demographic features.

We define LSTM-C-all as the set of positive ICU readmission cases which can only be identified by LSTM+CNN model and not any of the six baseline regression models as mentioned above. Overall, 201 cases are contained in LSTM-C-all.

Furthermore, we define the following sets: (i) LSTM-LR-L1-B_STAT set, (ii) LSTM-LR-L1-A_STAT set, (iii) LSTM-LR-L1-A_STAT-D set, (iv) LSTM-LR-L2-B_STAT set, (v) LSTM-LR-L2-A_STAT set, (vi) LSTM-LR-L2-A_STAT-D set as the sets that are correctly predicted by both the LSTM+CNN and respective baseline logistic regression models. Summary of the number of cases contained in each of these sets is shown in Table 6.

**Table 6:** Summary of the number of cases correctly predicted by the corresponding baseline model as well as the LSTM+CNN.

Name of the set	Number of cases
LSTM-C-all	201
LSTM-LR-L1-B_STAT	3,033
LSTM-LR-L1-A_STAT	3,022
LSTM-LR-L1-A_STAT-D	3,068
LSTM-LR-L2-B_STAT	3,044
LSTM-LR-L2-A_STAT	3,079
LSTM-LR-L2-A_STAT-D	3,084

**Fig 11:**
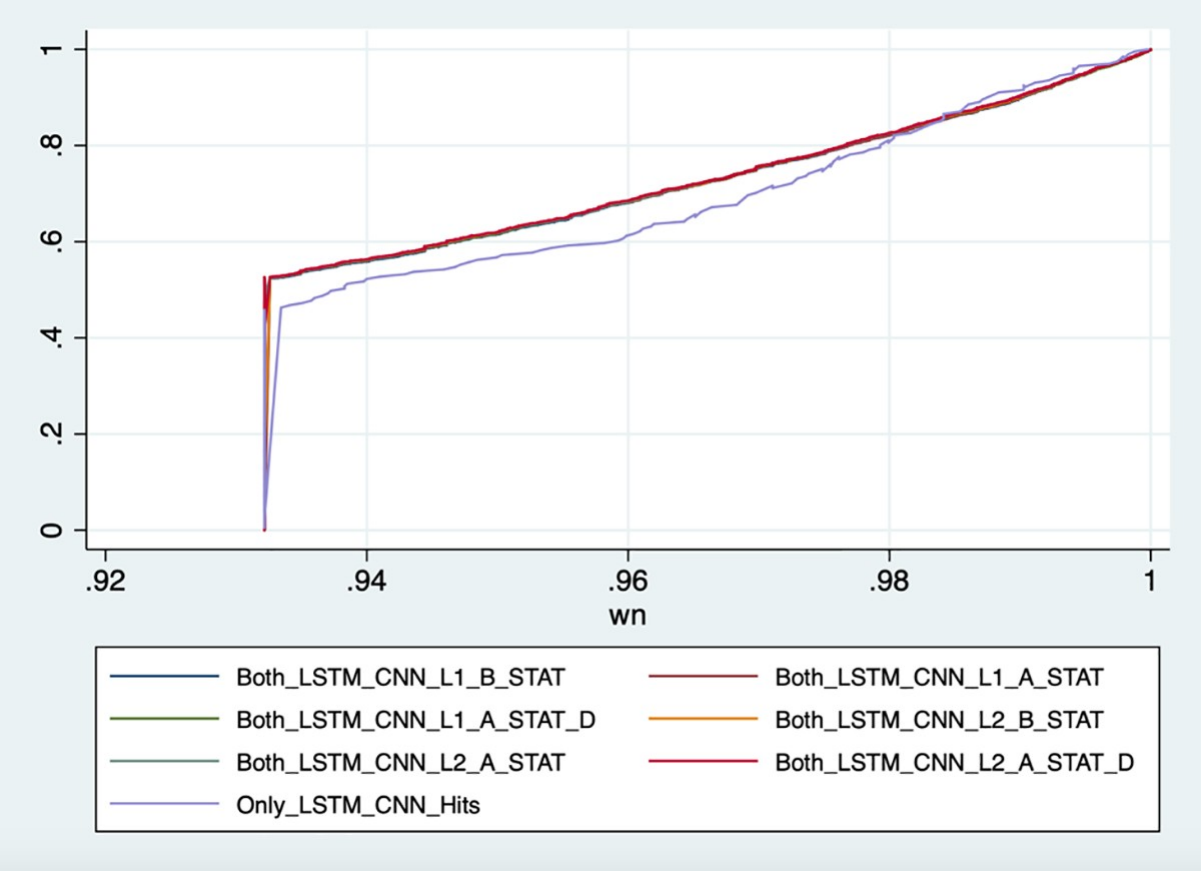
CDF plots of *w*_*n*_ for sets in Table 6.

We follow the same logic described in the previous section, to capture the maximum probability *w*_*n*_ of record *n* for each set mentioned above. Then, we plot the CDF of *w*_*n*_ for each set. Results are shown in Fig 11.

From Fig 11, we can observe that the CDF representing oscillation of the LSTM-C-all set is still the lowest one. The observation is consistent with the previous observation in the section “Strengths of the model”: there are more patient records in the LSTM-C-all which have at least one chart event with high oscillation sequence. The result enhances our argument that compared to baseline logistic regression models, our LSTM+CNN model is capable of capturing high volatile time series behavior, a common pattern in high-risk ICU patients.

The rest of the CDF lines represent oscillation of the six sets predicted correctly by both the LSTM+CNN and various logistic regressions models. We observe that the six CDFs are almost identical. Based on this observation, we conclude that even though using A_STAT (mode advanced statistical features) can slightly enhance the performance of baseline logistic regression models (as shown in Table 3), it can hardly improve the ability of logistic regressions to capture the critical oscillations in ICU patients. The results further enhance the advantage of using LSTM based model to identify patients with a high risk of readmission in a critical department such as ICU.

## Conclusion

In this study, we addressed the unplanned ICU readmission prediction by utilizing chart events, demographics and ICD-9 embeddings features. Among the data that we used, chart event features are significantly sensitive to time series, and cannot be properly captured by conventional machine learning models (e.g., logistic regression). We propose a LSTM-CNN based model, which can properly incorporate time series data without information lost.

Our machine learning solution for prediction ICU readmission offers higher accuracy and sensitivity compared to existing solution. In addition, since the model can have multiple operating points, its sensitivity and specificity can be tuned to match requirements for specific clinical settings, such as high sensitivity for critical care. In this study, AUC of 0.791 and sensitivity of 0.742 were achieved. Moreover, we illustrated the importance of each input features and their combinations in the predictive model This fast and interpretable solution holds the potential for substantial clinical impact by augmenting clinical decision-making for ICU specialists. Further research is necessary to evaluate performance in a real-world, clinical setting, in order to validate this technique across varying critical care practices.
